# Supplementary material for: Chromothripsis during telomere crisis is independent of NHEJ, and consistent with a replicative origin
Source: Genome Res. 2019 May;29(5):737–49. doi: 10.1101/gr.240705.118 (PMC6499312; doi:10.1101/gr.240705.118)
Supplement: Supplemental Material [file supp_gr.240705.118_Supplemental_file_1.zip › contigs/annotated_contigs/DB53/contig.2.DB53_length_963_mean_cov_16.8255451713.docx]

**DB53_length_963_mean_cov_16.8255451713**

```
ATTAAGTATCCATGTTACAGTCCTAATCTGGATCCTTCTGACTTCTTTTTGTTTTCTGATCCTAAAACAAAAAATTTTAAGGCACCTAT
 >chr17:68549330-68549774 - E=4e-252 p=4e-02
TTTTCTTCAGCTAATATGGAAAAAACACTGCATTGACATGGTTAAATTCCAAGGACCATCGGTTTTTTATAGGTGGACTAAATGGCAGG

TATCATCACTTACAAAAGTGTCCTGAACTTGAGGAAGCTTATGTTGACAAAGCTTGTATTTTTAATTTTTATCTTTAATTCAATTTTTC

TCTGAAATTTTTATCTAAGTCCTCTTGCATATGTCATACACATATATCTATAAGACTTAATCAGAACTCTAATTTTATGGAATCTAGGT

CCTTAAAATCTGTTGCTTAAGGAACATCTAATCTTCATTGAAGTGCCCTTTTGTTCCTGGATTCCTGTTCTTTGTTAACAGTGTCAGT|

GACACT|GGATGAATGAAGAGCCCAAGAATGAACATTTATGTAGCATGCCACTACCTGCCAGTCATCAGAGCCACCCTGGTGTATAAAG
      >chr8:85467199-85467712 - E=1e-290
CAGAGGTTTCCCATATTGTCATGGAAATTACTGTCTAGATGAGGAGAGAAGTGTTAAACAAGTAATCATGTGGGAGCCAACATATATAT

ATGTGTGTGTGTGTGTGTATGTATATATTGAGATGGAGTCTCACTCTGTTGCCACGTTGGAGTGCAGTGGCATGATCTCGGCTCACTGC

AACCTCCGCCTCCCGGGTTCAAGCGATTCTCCTGCCTCAGCCTCCTGAGTAGCTGGGATTACAGGCGTGTGCCACCACGCCCGGCTAAT

TTTTGTATTTTTAGTAGAGGTGGGGTTTCACCATGTTGGTCAGGCTGGTCTTGAACGCCTGACCTCGTGATCTGCCCGCCTTAGCCTCC

CAAAGTGCTGGGATTACAGGCGTGAGCCACTGTGCCAGGCCAACATATTTTATAGCTAGGATCTAAAGTCAGCAT
```
